# Co-injection with inactivated *Enterococcus faecium* and inactivated H1N1 influenza virus intravenously strengthen the protection of H1N1 influenza virus infections in mice

**DOI:** 10.3389/fmicb.2025.1641008

**Published:** 2025-08-20

**Authors:** Jinlian Li, Yifei Ren, Mei Xue, Di Shao, Lili Zhuang, Zhengyang Shen, Zitong Tang, Yuan Cui, Youfei Shi

**Affiliations:** ^1^College of Biology and Brewing Engineering, Taishan University, Tai’an, China; ^2^Tecon Biology Co., Ltd., Ürümqi, China; ^3^College of Animal Science and Veterinary Medicine, Shandong Agricultural University, Tai’an, China

**Keywords:** *Enterococcus faecium*, H1N1 influenza virus, immunization, intravenous, adjuvant

## Abstract

Influenza viruses pose a significant threat to human and animal health globally. Vaccine immunization is an effective strategy for preventing disease, reducing morbidity and economic losses, and enhancing quality of life. *Enterococcus faecium* is a Gram-positive, facultative anaerobic, lactic acid-producing bacterium that resides as a commensal in the gastrointestinal tract of animals and serves as a probiotic. This study investigated the effects of intravenous and intramuscular administration of inactivated *E. faecium* and inactivated influenza A H1N1 (PR8) virus on body weight, lung histopathology, HI antibody titers, immune cell composition in the spleen, and cytokine expression and viral load in the lungs of experimental mice following challenge. The results demonstrated that intravenous co-administration of inactivated *E. faecium* and inactivated H1N1 significantly mitigated weight loss and was associated with increased proportions of B cells, CD8^+^ T cells, and macrophages in the mouse spleen compared to other groups. Histopathological analysis revealed enhanced vascular-centered immune responses in the lungs of mice co-administered with inactivated *E. faecium* and inactivated H1N1. These findings suggest that co-administration of inactivated *E. faecium* and H1N1 virus enhances protection against H1N1 infection in mice, potentially improving vaccine efficacy.

## Introduction

Influenza is a highly contagious acute respiratory disease with significant morbidity and mortality rates in both humans and animals (Kemble and Greenberg, 2003). Influenza viruses (IVs) pose a major threat to human health and the global economy (Sun et al., 2013). IVs belong to the Orthomyxoviridae family, consisting of enveloped viruses that contain eight single-stranded RNA segments. These viruses are categorized into four types: A, B, C, and D. Among them, influenza A virus (IVA) is the most virulent in humans. Only IVA has the potential to cause zoonotic infections, host shifts, and pandemics (Ghafoori et al., 2023; Hause et al., 2014; Taubenberger and Kash, 2010; Wang et al., 2022). IVA viruses are clinically the most significant as they can induce severe diseases in multiple species, including humans, pigs, birds, horses, cattle, whales, seals, tigers, dogs, cats, and ferrets (Li and Robertson, 2021; Mehle, 2014). Annually, approximately 10% of the global population is infected with IVs, resulting in 300,000 to 600,000 deaths worldwide and healthcare costs exceeding USD 87 billion (Iuliano et al., 2018; Nickol and Kindrachuk, 2019). The H1N1 influenza virus, a subtype within the AIV, has emerged repeatedly and unpredictably throughout modern history (Sharon and Schleiss, 2009). Vaccination remains the most effective strategy for preventing IV infections and their associated severe complications (Chen et al., 2020; Grohskopf et al., 2022; Houser and Subbarao, 2015).

Vaccine adjuvants play a critical role in modulating both innate and adaptive immune responses, thereby reducing the required antigen doses and the number of immunizations (Boyle et al., 2007; Reed et al., 2013). Probiotics have been shown to enhance animal growth and performance (Ahiwe et al., 2020), contribute to maintaining immune homeostasis (Galdeano and Perdigón, 2006), and act as effective adjuvants to augment the immunogenicity and efficacy of live vaccines (Soltani et al., 2019). Additionally, probiotic bacteria exhibit an excellent safety profile and function as antigen delivery systems. They stimulate cytokine release from macrophages and T cells, induce antibody production, and improve T cell-mediated immune responses (Song et al., 2012; Shi et al., 2014; Ma et al., 2018; Hayashi et al., 2018; Hong et al., 2019; Li et al., 2019; Maldonado Galdeano et al., 2019; Nouri Gharajalar et al., 2020; Lee et al., 2020).

Vaccines are delivered through various routes, including intramuscular, subcutaneous, intradermal, nasal, and oral administration. Intravenous vaccine immunization has been utilized since the 1960s and has demonstrated efficacy. For example, studies have shown that intravenous administration of BCG in mice and rhesus monkeys enhances resistance to *Mycobacterium tuberculosis* infections and reduces pulmonary tissue lesions (Anacker et al., 1969; Barclay et al., 1970; Darrah et al., 2020). Additionally, a study reported that primary vaccination with the recombinant vaccinia virus V25, expressing the full-length gp160 env protein of the HIV strain HTLV-IIIB, elicited a weak immune response. This response was significantly improved using four different immunization protocols, with the intravenous administration of paraformaldehyde-fixed autologous cells infected *in vitro* with V25 yielding the most favorable results (Zagury et al., 1988). Conversely, a separate study reported no significant differences in hematological and coagulation parameters, histopathological characteristics, innate immune responses, or the risk of vaccine-induced immune thrombotic thrombocytopenia (VITT) between intravenous and intramuscular administration of the Ad26.COV2.S vaccine in rabbits (Khan et al., 2023). In another experiment, mice that received intravenous injections of Bacillus Calmette–Guérin (BCG) demonstrated effective protection against influenza A virus infections, as well as enhanced viral clearance when challenged with either the SARS-CoV-2 B.1.351 variant or the H1N1 (PR8) influenza virus. This cross-protection effect mediated by BCG is attributed to the enrichment of CD4^+^ effector CX3CR1hi memory αβ T cells in both the bloodstream and lung parenchyma. Lung-resident CX3CR1hi T cells were found to reduce early viral infection through the production of IFN-γ (Lee et al., 2024; Tran et al., 2024). Furthermore, the intravenous delivery of attenuated Salmonella or a self-assembling nanoparticle vaccine incorporating neoantigen peptides linked to a Toll-like receptor 7/8 agonist significantly enhanced anti-tumor immune responses (Yi et al., 2020; Baharom et al., 2021).

We previously demonstrated that the intravenous administration of inactivated *E. faecium* and inactivated pseudorabies virus resulted in elevated antibody production, increased levels of CD4^+^ and CD8^+^ cells, and reduced mortality in mice infected with live pseudorabies virus compared to control groups (Cui et al., 2023). This study investigates the effects of both intravenous and intramuscular administrations of inactivated *E. faecium* and influenza A (H1N1) virus on body weight, lung histopathology, HI antibody titers, types and percentages of immune cells, cytokine expression, and viral load in the lungs of mice. These findings may contribute to enhancing vaccine development and efficacy.

## Materials and methods

### Bacterial and viral strains and mice

The influenza A/Puerto Rico/8/34 (H1N1) virus was maintained by the laboratory. The *E. faecium* CICC 6049 strain was obtained from the China Center of Industrial Culture Collection. Specific-pathogen-free Kunming mice weighing 18–22 g were procured from Shandong Taibang Biological Products Co., Ltd. (Tai’an, China). Following immunization, the mice were provided with ad libitum access to standard chow and water for a period of 7 days.

### Preparation of inactivated H1N1 virus

The H1N1 virus was propagated in 10-day-old embryonated chicken eggs and subsequently purified via centrifugation in a discontinuous sucrose gradient to yield ultra-pure viral particles. The median egg infectious dose (EID_50_) of the viral solution was quantified as 10^8^EID_50_/0.1 mL. The purified virus was chemically inactivated using formaldehyde at a final concentration of 0.3%. Subsequently, the inactivated virus was inoculated into a monolayer of MDCK cells cultured in three T-flasks and incubated at 37°C for 7 days; no cytopathic effects were observed during this period. Sterility testing was performed by culturing the viral solution in bouillon and agar media at 37°C for 24–48 h, with no bacterial growth detected. For safety evaluation, 10 mice were subcutaneously inoculated with 200 μL of the inactivated viral solution and monitored daily for 14 days. Unvaccinated mice served as controls. No local or systemic adverse reactions attributable to vaccination were observed throughout the observation period.

### Experimental design

The animal study protocol was approved by the Animal Care and Use Committee (ACUC) of Shandong Agricultural University (Protocol No. SDAUA-2024-056). The study strictly followed the guidelines established by the committee. All experiments were conducted in accordance with the Guide for the Care and Use of Laboratory Animals issued by the Ministry of Science and Technology of China. Following a 3-day acclimatization period, 60 4-week-old mice were randomly divided into six groups, each consisting of 10 mice, and treated as described in Table 1. Three days after inoculation, all groups except the negative control were challenged with 150 μL of live H1N1 virus (10^8^EID_50_/0.1 mL) via nasal instillation. The negative control group received an equivalent volume of saline via the same route. Behavioral changes, including lethargy, hunched posture, curling behavior, ruffled fur, trembling, delayed responsiveness, reduced activity, and clustering, were monitored daily. Mice were humanely euthanized at 5 days post-infection (dpi). Mice exhibiting extreme lethargy were deemed moribund and were excluded from further experimentation.

**Table 1:** Method of immunization in each group.

Group	Method of immunization
Negative control group (Group 1)	Intramuscular administration of 0.2 mL of saline
Positive control group (Group 2)	Intramuscular administration of 0.2 mL of saline
Intravenously injected with inactivated *E. faecium* (Group 3)	Intravenous administration of 0.2 mL of inactivated *E. faecium*
Intravenously injected with inactivated H1N1 virus and saline group (Group 4)	Intravenous administration of 1:1 mixture of inactivated H1N1 virus and saline 0.2 mL
Intravenously injected with inactivated *E. faecium* + inactivated H1N1 virus group (Group 5)	Intravenous administration of a 1:1 mixture of inactivated *E. faecium* and inactivated H1N1 virus 0.2 mL
Intramuscularly injected with inactivated *E. faecium* + inactivated H1N1 virus group (Group 6)	Intramuscular administration of a 1:1 mixture of inactivated *E. faecium* and inactivated H1N1 virus 0.2 mL

### Measurements of body weight gain

Body weight was measured daily for seven consecutive days. The body weight change curve for mice in each group over the 7-day period was generated. The body weight of the mice at day 1 post-infection (dpi) was subtracted from their body weight at 7 dpi, and a histogram representing the distribution of body weight gains was constructed. Additionally, the average weight gain was calculated. Mice that lost ≥25% of their initial body weight were considered moribund and were humanely euthanized.

### Histopathological examination

During necropsy, the lungs of mice were harvested, fixed in 4% phosphate-buffered formalin, embedded in paraffin, serially sectioned at a thickness of 2 to 4 μm, and subsequently stained with hematoxylin and eosin. Histopathological evaluation was conducted by an experienced pathologist who was blinded to the group allocation.

### Measurement of serum HI antibody titers

H1N1-specific HI antibodies at 5 days post-infection (dpi) were quantified using hemagglutination inhibition (HI) assays. Briefly, sera were pretreated with receptor-destroying enzyme (RDE; Denka Seiken, Tokyo, Japan) according to the manufacturer’s protocol and then adsorbed with a 20% suspension of chicken red blood cells (RBCs) for 1 h at 4°C. For each virus used in the HI assay, it must be standardized to contain 4 HA units/25 μL or 8 HA units/50 μL. The treated serum (1:10) was serially diluted two-fold with PBS. Then, 25 μL of standardized virus antigen were added into 25 μL of the serial two-fold dilutions of antisera (1:10–1:1,280) and the mixtures were incubated for 30 min at 37°C. Finally, 50 μL of 1% RBCs were added into 50 μL of the mixture. After incubation at room temperature for 30 min, HI titres were determined as the reciprocal of the highest dilution of the serum inhibiting agglutination (showing RBC button).

### Flow cytometry

At 1, 3, and 5 days post-infection (dpi), spleens were harvested and transferred to 24-well plates. Subsequently, 1 mL of RPMI 1640 culture medium was added to each well. The medium was aspirated, and 1 mL of trypsin solution was added to each well. Spleens were minced using sterile scissors and incubated at 37°C for 30 min. Trypsin digestion was terminated by adding 1 mL of phosphate-buffered saline (PBS). The spleen tissues were further dissociated and filtered through a 100 μm cell strainer. The cell suspension was adjusted to a final volume of 10 mL with PBS and centrifuged at 2,500 rpm for 6 min at 4°C. The supernatant was discarded, and the pellet was resuspended in 1 mL of PBS, filtered again through a cell strainer, and divided into two aliquots. For flow cytometry analysis, 30 μL of the cell suspension was incubated with fluorophore-conjugated antibodies against mouse CD3e (FITC), CD4 (Alexa Fluor 488), CD8a (PE), CD64 (PE), F4/80 (FITC), and human/mouse CD45R (B220; PerCP-Cyanine 5.5) for 15 min at room temperature. The percentages of immune cell subsets were quantified using flow cytometry. Data were analyzed using FlowJo software version 10.10.

### Reverse transcription-quantitative polymerase chain reaction

At 1, 3, and 5 days post-infection (dpi), lungs were harvested, and cytokine expression levels as well as viral loads were quantified using quantitative real-time PCR (qRT-PCR). Total RNA was isolated with the RNeasy RNA extraction kit (Qiagen) and reverse-transcribed into cDNA using the Evo M-MLV Reverse Transcription Kit (Accurate Biology Co., Ltd., Changsha, China). Quantitative PCR was performed using the SYBR Green Pro Taq HS qPCR Kit (Accurate Biology) in a reaction mixture containing 10 μL of 2 × SYBR Green Premix, 1 μL of template cDNA, 0.4 μL of each primer, and 8.2 μL of RNase-free water. The thermal cycling conditions consisted of an initial denaturation step at 95°C for 30 s, followed by 40 cycles of denaturation at 95°C for 5 s, annealing at 60°C for 30 s, and extension at 95°C for 10 s. A final melting curve analysis was conducted from 65°C to 97°C. Primer sequences are listed in Table 2 (primers were designed based on gene sequences retrieved from GenBank using Primer Premier 5.0 software and synthesized by Hunan Aikerui Bioengineering Co., Ltd.). The resulting Ct values were substituted into the standard curve for reverse transcription-quantitative polymerase chain reaction (RT-qPCR) analysis. The viral copy number in the lungs was calculated using the standard equation (*y* = −3.082*x* + 35.272, *R*^2^ = 0.9992). The relative expression levels of target genes encoding various cytokines in mice across all groups were determined using the 2^−ΔΔCq^ method.

**Table 2:** Primer sequences used in PCR.

Genes	Forward sequence	Reverse sequence
NP	GCCAGTGGGTACGACTTTGA	CTCTTGGGACCACCTTCGTC
IL-1β	TCCAGGATGAGGACATGAGCAC	GAACGTCACACACCAGCAGGTTA
IL-10	GCCAGAGCCACATGCTCCTA	GATAAGGCTTGGCAACCCAAGTAA
IFN-γ	CGGCACAGTCATTGAAAGCCTA	GTTGCTGATGGCCTGATTGTC
TNF-α	GCCAGGAGGGAGAACAGAAACTC	GGCCAGTGAGTGAAAGGGACA
β-actin	CATCCGTAAAGACCTCTATGCCAAC	ATGGAGCCACCGATCCACA

### Statistical analysis

Statistical analysis was conducted using SPSS version 27.0 (IBM Corp., Armonk, NY) and GraphPad Prism version 10 (GraphPad Software, Inc., La Jolla, CA). Continuous variables that followed a normal distribution were presented as means ± standard deviations. The significance of differences among group means was assessed using one-way analysis of variance (ANOVA). All statistical tests were two-tailed, and *p*-values less than 0.05 were considered to indicate statistical significance.

## Results

### Clinical signs and changes in body weight

**Figure 1:**
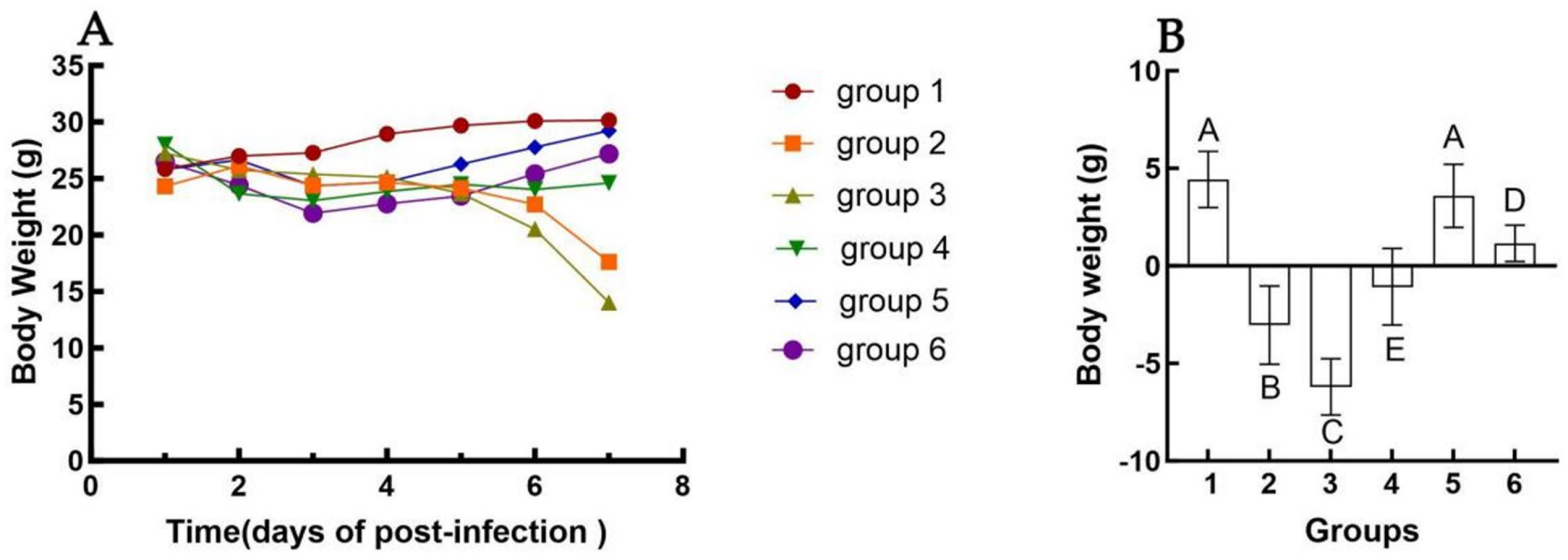
Changes in body weight of different groups after challenge. The weight change of mice in each group from 1 dpi to 6 dpi **(A)**. The difference in body weight of mice in each group from the day of challenge to 6 dpi **(B)**. Group 1 was the negative control. Group 2 was the positive control. Group 3 was intravenously injected with inactivated *E. faecium*. Group 4 was intravenously injected with inactivated H1N1 virus and saline. Group 5 was intravenously injected with inactivated *E. faecium* + inactivated H1N1 virus. Group 6 was intramuscularly injected with inactivated *E. faecium* + inactivated H1N1 virus. Different lowercase letters indicate significant differences at *p* < 0.05, while different uppercase letters indicate highly significant differences at *p* < 0.01. When comparing differences between groups, first examine the uppercase letters. If the uppercase letters differ, this indicates *p* < 0.01, and there is no need to compare the lowercase letters. If the uppercase letters are the same, it is necessary to further compare the lowercase letters. If the lowercase letters differ, this signifies *p* < 0.05; otherwise, it indicates no significant difference.

Changes in body weight following immunization are presented in Figure 1. Mice exhibited a normal physiological response post-inoculation, with no significant alterations in feeding or drinking behavior observed. Starting from day 2 post-challenge, some mice in Groups 2 and 3 displayed clinical symptoms, including ruffled fur, hunched posture, and labored breathing. Inoculation with inactivated *E. faecium* or inactivated H1N1 resulted in a slight reduction in body weight up to day 4 (Figure 1A). A more pronounced decrease in body weight was observed in Groups 2 and 3. Conversely, body weight recovery was noted in Groups 4, 5, and 6, particularly in Group 5 (Figure 1B). No statistically significant difference in body weight changes was detected between Groups 1 and 5 at 6 days post-infection (dpi) (*p* > 0.05). However, a highly significant difference in body weight was observed between Groups 4 and 6 at 6 dpi (*p* < 0.01).

### Histopathological examination

**Figure 2:**
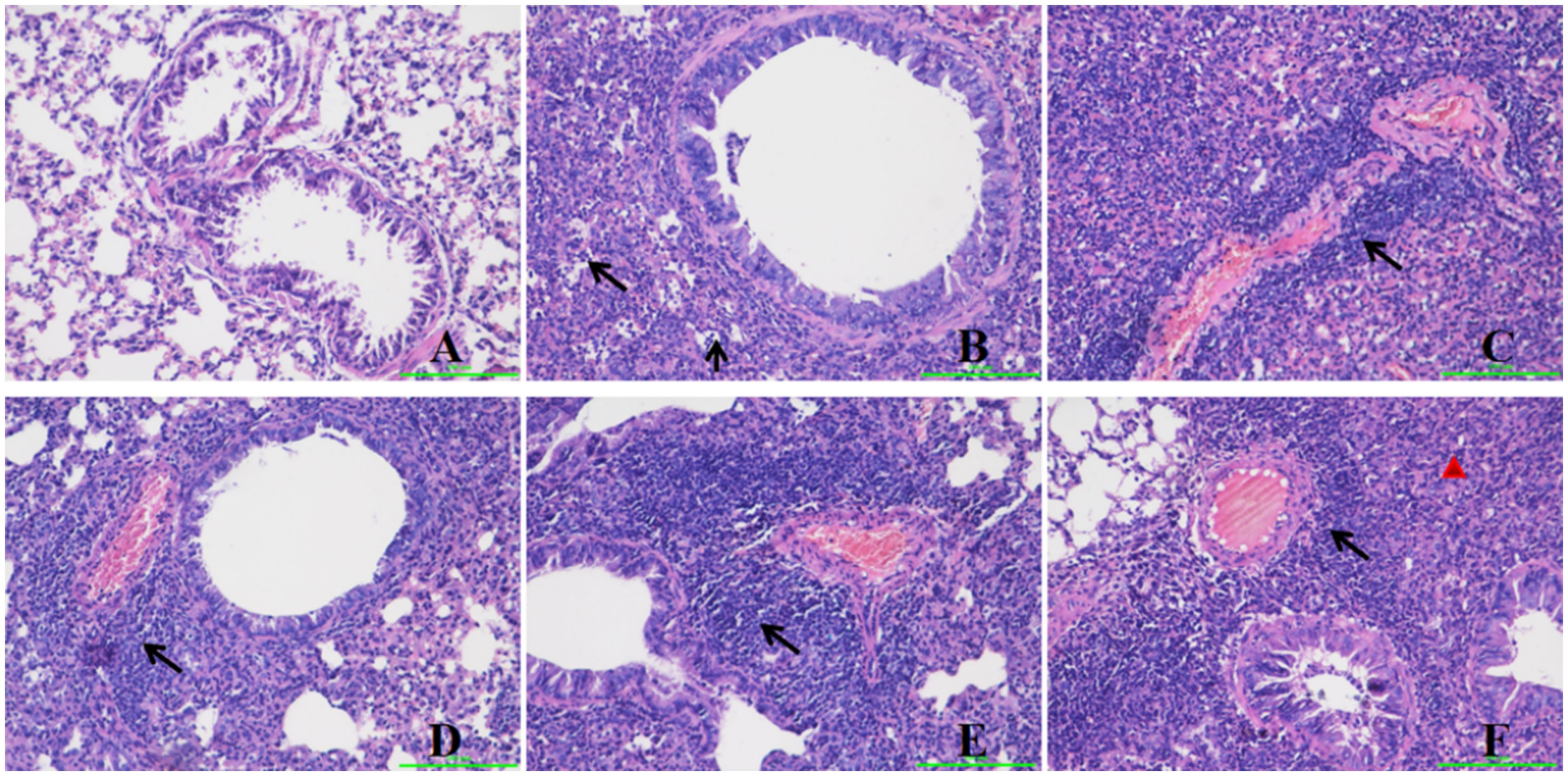
Histopathological changes in the mice lung after challenge. Group **A** was the negative control. Group **B** was the positive control. Group **C** was intravenously injected with inactivated *E. faecium*. Group **D** was intravenously injected with inactivated H1N1 virus and saline. Group **E** was intravenously injected with inactivated *E. faecium* + inactivated H1N1 virus. Group **F** was intramuscularly injected with inactivated *E. faecium* + inactivated H1N1 virus. Lung tissues were stained with hematoxylin and eosin (magnification, 200×). The triangle represents inflammatory cell infiltration. The arrows show lymphocyte infiltration.

Histopathological changes in the mouse lung at 5 days post-infection (dpi) are depicted in Figure 2. The negative control group exhibited no significant histological alterations. Conversely, several histopathological changes were observed in the lungs of immunized mice, including lymphocyte infiltration surrounding blood vessels, increased alveolar wall thickness, and inflammatory cell infiltration within the alveolar spaces (Figure 2B). Group 3 demonstrated pronounced lymphocyte infiltration around blood vessels, a marked increase in alveolar wall thickness, and mild inflammatory cell infiltration in the alveolar spaces (Figure 2C). Group 4 exhibited robust lymphocyte infiltration around blood vessels and a slight increase in alveolar wall thickness, with no evidence of red blood cells, alveolar fluid, or inflammatory cell infiltration in the alveolar spaces (Figure 2D). Group 5 showed intense lymphocyte infiltration around blood vessels and a significant increase in alveolar wall thickness, with no red blood cells, alveolar fluid, or inflammatory cell infiltration in the alveolar spaces (Figure 2E). Group 2 presented with mild lymphocyte infiltration around blood vessels, a marginal increase in alveolar wall thickness, and mild inflammatory infiltration in the alveolar spaces. Group 6 displayed a notable increase in alveolar wall thickness but lacked red blood cells, alveolar fluid, or inflammatory cell infiltration in the alveolar spaces (Figure 2F).

### HI antibody titers

**Figure 3:**
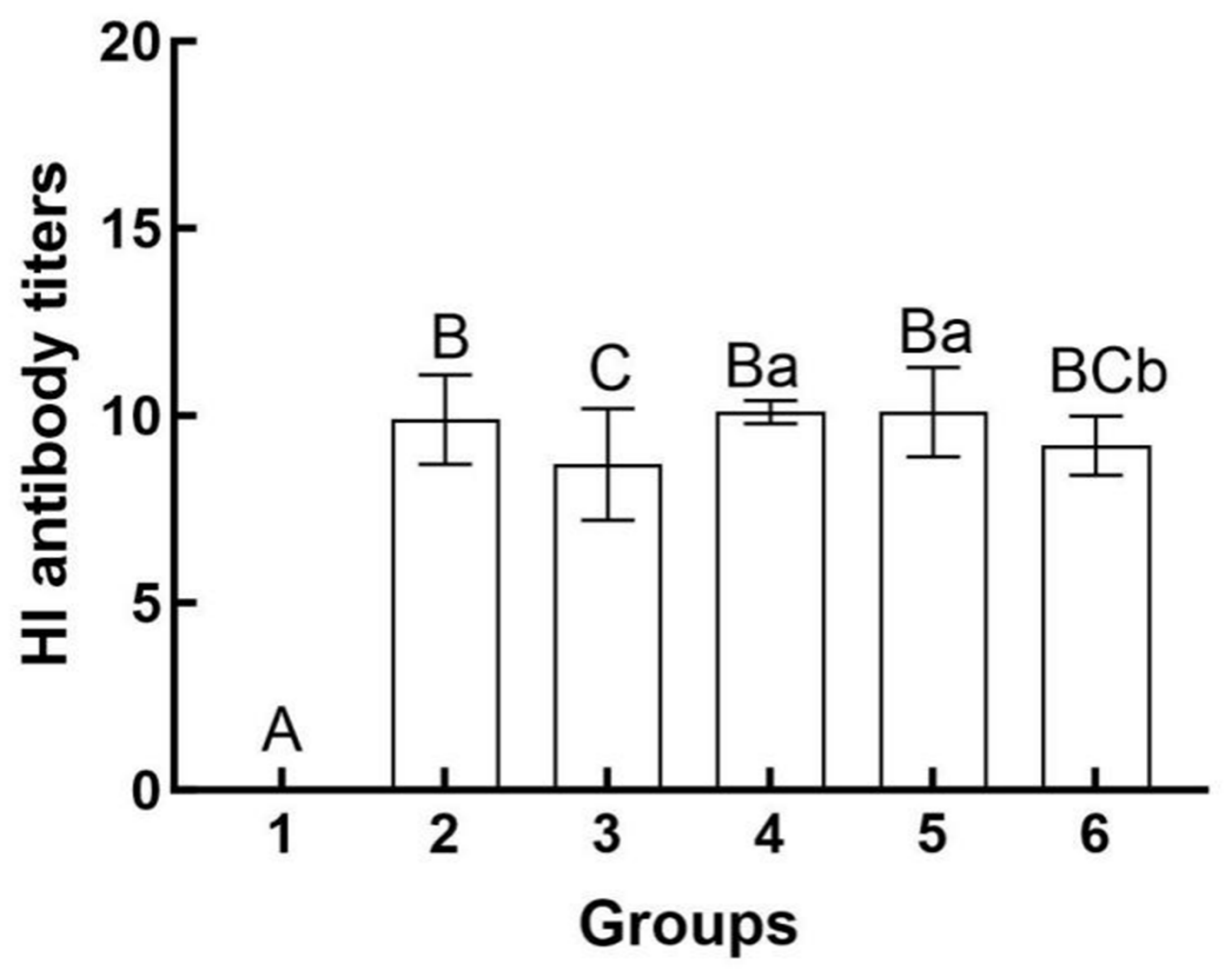
Hemagglutination inhibition antibody titers in different groups after challenge. Group 1 was the negative control. Group 2 was the positive control. Group 3 was intravenously injected with inactivated *E. faecium*. Group 4 was intravenously injected with inactivated H1N1 virus and saline. Group 5 was intravenously injected with inactivated *E. faecium* + inactivated H1N1 virus. Group 6 was intramuscularly injected with inactivated *E. faecium* + inactivated H1N1 virus. Different lowercase letters indicate significant differences at *p* < 0.05, while different uppercase letters indicate highly significant differences at *p* < 0.01. When comparing differences between groups, first examine the uppercase letters. If the uppercase letters differ, this indicates *p* < 0.01, and there is no need to compare the lowercase letters. If the uppercase letters are the same, it is necessary to further compare the lowercase letters. If the lowercase letters differ, this signifies *p* < 0.05; otherwise, it indicates no significant difference.

The results of the hemagglutination inhibition (HI) assays are presented in Figure 3. The serum HI antibody titers for mice in Group 4 ranged from 1:2^10^ to 1:2^11^, while those for Group 5 ranged from 1:2^9^ to 1:2^12^. The serum HI antibody titers for Group 6 were observed to range from 1:2^8^ to 1:2^10^. Notably, Groups 4 and 5 exhibited significantly higher serum HI antibody titers compared to Group 6 (*p* < 0.05). Conversely, no significant difference was detected between Groups 4 and 5 (*p* > 0.05). The disparity in efficacy between the two inoculation methods (intramuscular versus intravenous) is illustrated.

### Flow cytometry analysis

**Figure 4:**
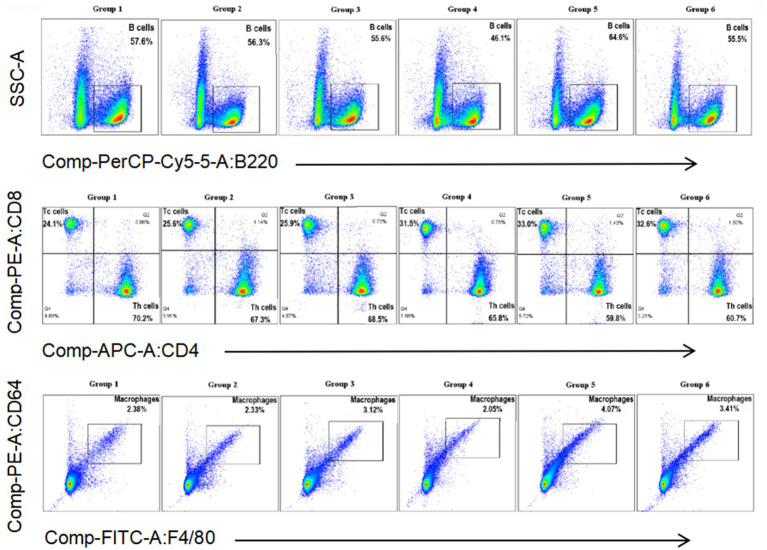
Percentage of B cells, Th cells, Tc cells, and macrophages in the spleen of mice from different groups at 5 days post-infection.

**Figure 5:**
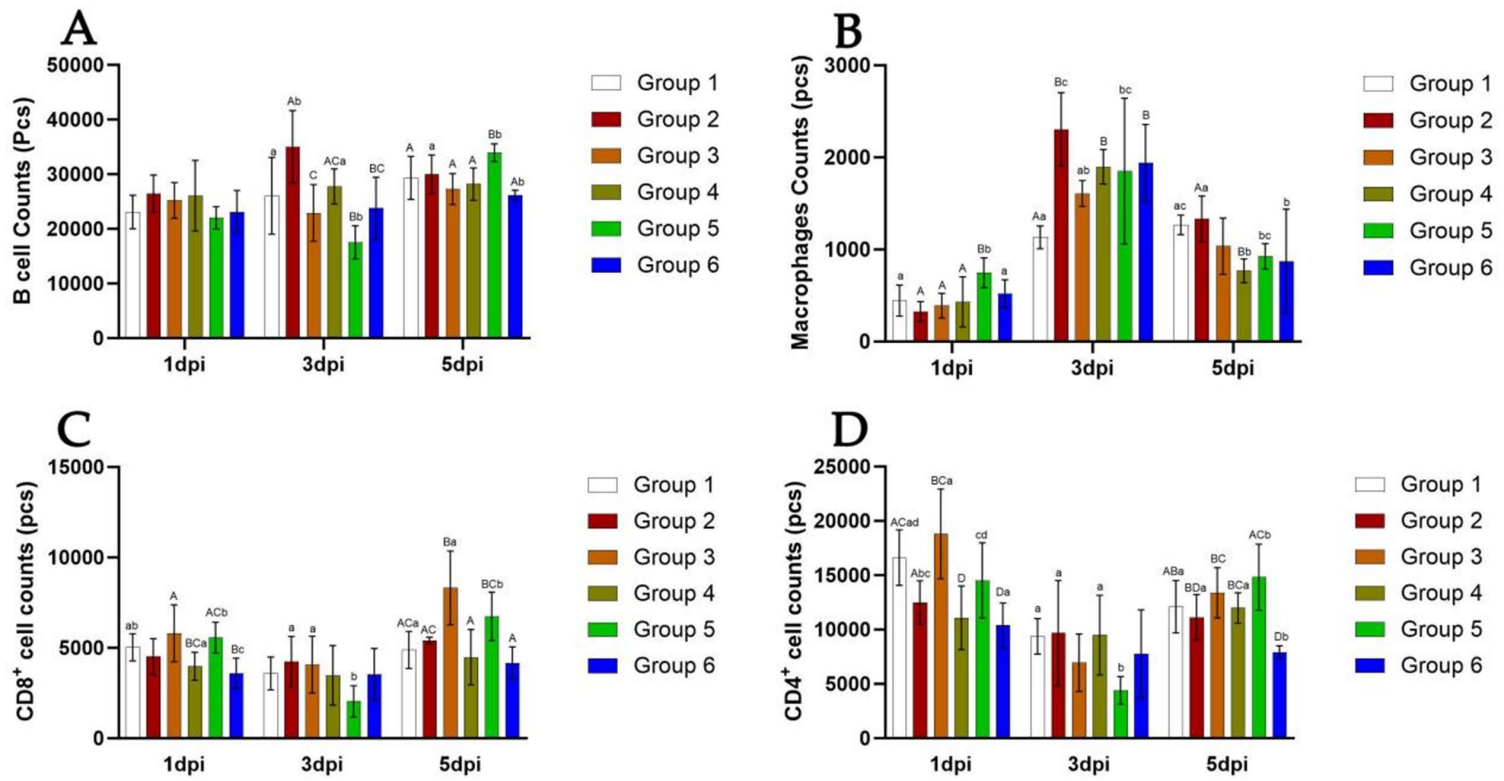
Number of B cells, Th cells, Tc cells, and macrophages in the spleen of mice from different groups at 5 days post-infection. The detection results of B cell number in each group of mice from 1 dpi to 5 dpi **(A)**. The detection results of Macrophages number in each group of mice from 1 dpi to 5 dpi **(B)**. The detection results of CD8^+^ cell number in each group of mice from 1 dpi to 5 dpi **(C)**. The detection results of CD4^+^ cell number in each group of mice from 1 dpi to 5 dpi **(D)**. Group 1 was the negative control. Group 2 was the positive control. Group 3 was intravenously injected with inactivated *E. faecium*. Group 4 was intravenously injected with inactivated H1N1 virus and saline. Group 5 was intravenously injected with inactivated *E. faecium* + inactivated H1N1 virus. Group 6 was intramuscularly injected with inactivated *E. faecium* + inactivated H1N1 virus. Different lowercase letters indicate significant differences at *p* < 0.05, while different uppercase letters indicate highly significant differences at *p* < 0.01. When comparing differences between groups, first examine the uppercase letters. If the uppercase letters differ, this indicates *p* < 0.01, and there is no need to compare the lowercase letters. If the uppercase letters are the same, it is necessary to further compare the lowercase letters. If the lowercase letters differ, this signifies *p* < 0.05; otherwise, it indicates no significant difference.

Cytometric analysis is presented in Figures 4, 5. At 5 days post-infection (dpi), the percentages of B cells, CD8^+^ T cells, and macrophages were significantly higher in the spleen of mice from Group 5 (64.6, 33.0, and 4.07%, respectively) compared to other groups (Figure 4). Conversely, the percentage of CD4^+^ T cells was lower in this group (59.8%). Additionally, in Group 5, the counts of B cells, CD4^+^ T cells, and CD8^+^ T cells tended to decrease at 3 dpi, while the count of macrophages tended to increase at the same time point (Figure 5). At 5 dpi, the counts of B cells and CD8^+^ T cells were significantly higher in Group 5 compared to Groups 4 and 6 (*p* < 0.01) (Figures 5A,C), whereas the count of CD4^+^ T cells was significantly lower in Group 6 (*p* < 0.01) (Figure 5D). No significant intergroup differences were observed in the count of macrophages (Figure 5B).

### Cytokine expression analysis

**Figure 6:**
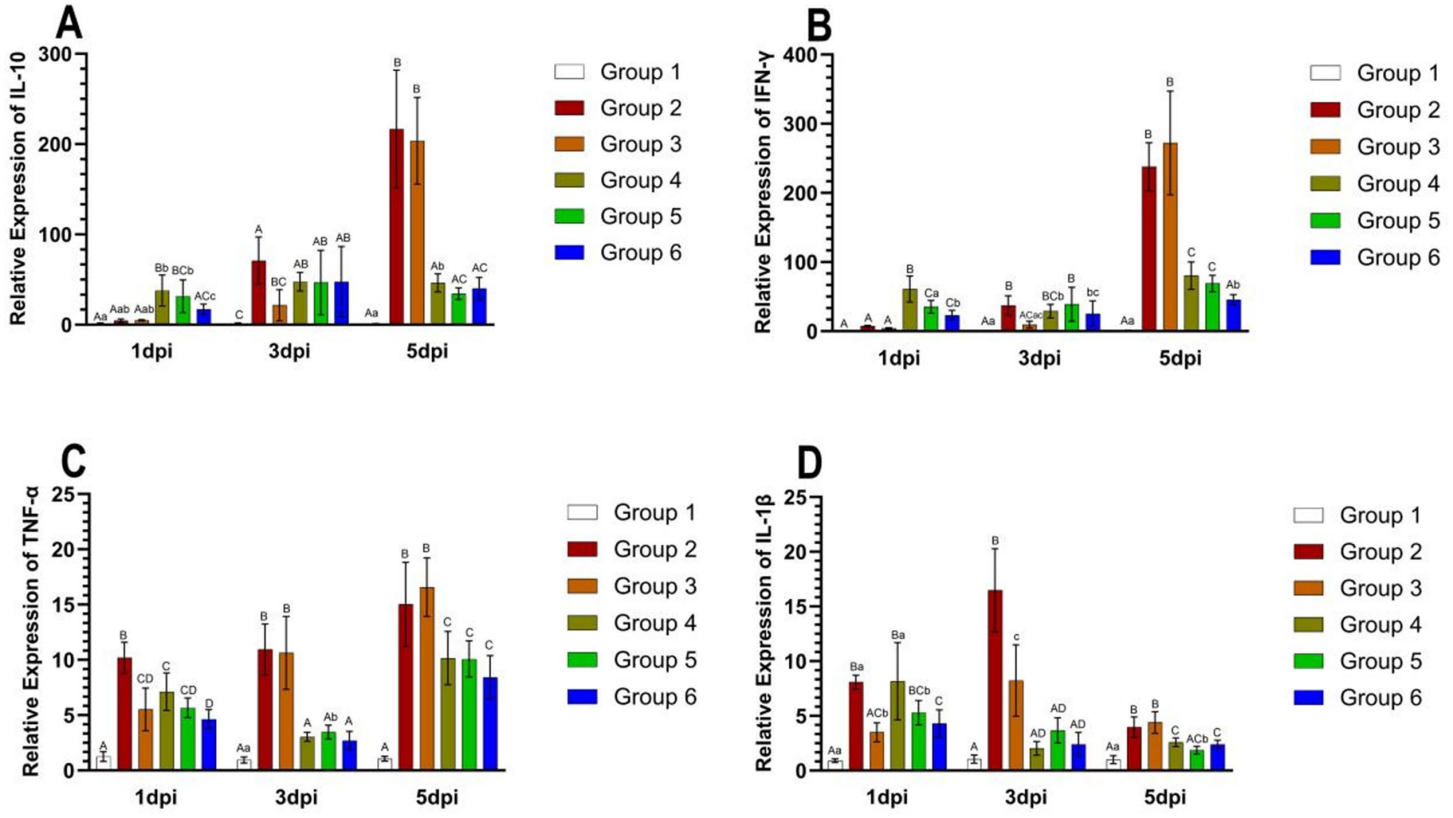
The relative expression levels of IL-10, IFN-γ, TNF-α, and IL-1β in the lungs of mice from different groups at 5 days post-infection. The expression of IL-10 in mice in each group from 1 dpi to 5 dpi **(A)**. The expression of IFN-γ in mice in each group from 1 dpi to 5 dpi **(B)**. The expression of TNF-α in mice in each group from 1 dpi to 5 dpi **(C)**. The expression of IL-1β in mice in each group from 1 dpi to 5 dpi **(D)**. Group 1 was the negative control. Group 2 was the positive control. Group 3 was intravenously injected with inactivated *E. faecium*. Group 4 was intravenously injected with inactivated H1N1 virus and saline. Group 5 was intravenously injected with inactivated *E. faecium* + inactivated H1N1 virus. Group 6 was intramuscularly injected with inactivated *E. faecium* + inactivated H1N1 virus. Different lowercase letters indicate significant differences at *p* < 0.05, while different uppercase letters indicate highly significant differences at *p* < 0.01. When comparing differences between groups, first examine the uppercase letters. If the uppercase letters differ, this indicates *p* < 0.01, and there is no need to compare the lowercase letters. If the uppercase letters are the same, it is necessary to further compare the lowercase letters. If the lowercase letters differ, this signifies *p* < 0.05; otherwise, it indicates no significant difference.

The relative expression levels of IL-10, IFN-γ, TNF-α, and IL-1β in the lungs are presented in Figure 6. The expression of IL-10 and IFN-γ demonstrated a tendency to increase over time in Groups 2 and 3. At 5 days post-infection (dpi), IL-10 expression was significantly elevated in Groups 2 and 3 compared to the other groups (*p* < 0.01) (Figure 6A). IFN-γ expression was slightly reduced in Group 4 at 3 dpi and slightly increased in Groups 5 and 6 at 5 dpi. Notably, IFN-γ expression was significantly higher in Groups 2 and 3 than in the other groups at 5 dpi (*p* < 0.01) (Figure 6B). TNF-α expression exhibited an increasing trend over time in Groups 2 and 3 and was significantly lower at 3 dpi in Groups 4, 5, and 6. Furthermore, TNF-α expression was significantly higher in Groups 2 and 3 at both 3 dpi and 5 dpi (*p* < 0.01) (Figure 6C). IL-1β expression tended to be higher at 3 dpi in Groups 2 and 3, whereas it showed a decreasing trend over time in the other groups (*p* < 0.01) (Figure 6D).

### Viral load

**Figure 7:**
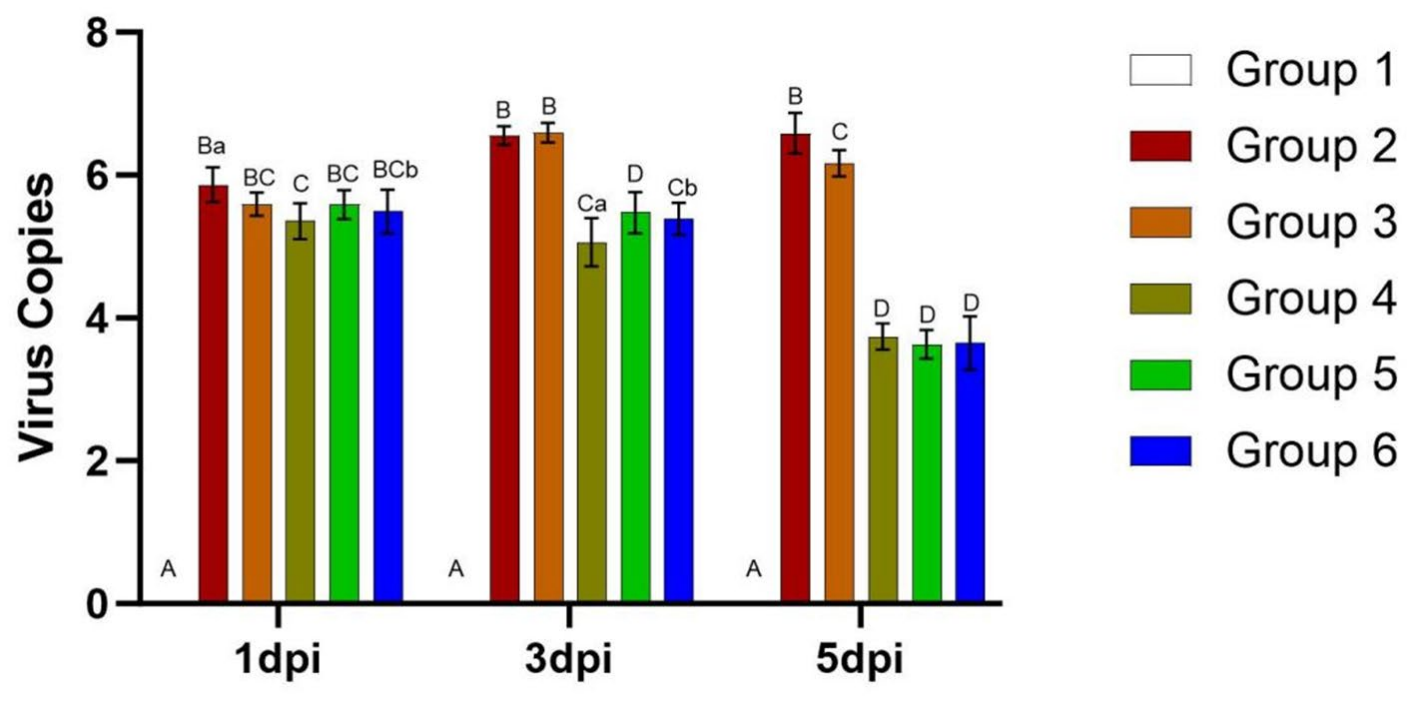
Viral loads in the lungs of mice from different groups after challenge. Group 1 was the negative control. Group 2 was the positive control. Group 3 was intravenously injected with inactivated *E. faecium*. Group 4 was intravenously injected with inactivated H1N1 virus and saline. Group 5 was intravenously injected with inactivated *E. faecium* + inactivated H1N1 virus. Group 6 was intramuscularly injected with inactivated *E. faecium* + inactivated H1N1 virus. Different lowercase letters indicate significant differences at *p* < 0.05, while different uppercase letters indicate highly significant differences at *p* < 0.01. When comparing differences between groups, first examine the uppercase letters. If the uppercase letters differ, this indicates *p* < 0.01, and there is no need to compare the lowercase letters. If the uppercase letters are the same, it is necessary to further compare the lowercase letters. If the lowercase letters differ, this signifies *p* < 0.05; otherwise, it indicates no significant difference.

Viral loads in the mouse lungs at 1, 3, and 5 days post-infection (dpi) are presented in Figure 7. At 3 and 5 dpi, viral loads were significantly higher in Groups 2 and 3 compared to the other groups (*p* < 0.01). In Groups 2 and 3, the viral load in the lungs of mice remained consistently high from 1 dpi to 5 dpi, ranging between 5.59 to 6.6. Conversely, viral loads decreased from day 3 to day 5 in Groups 4, 5, and 6, dropping from approximately 5.59 to around 3.63.

## Discussion

This study evaluated the protective efficacy of intravenous administration of inactivated *E. faecium* and inactivated H1N1 virus in mice. HI assays demonstrated that intravenous immunization elicited significantly higher HI antibody titers compared to intramuscular immunization. These findings provide a foundation for assessing the efficacy of various immunization approaches. Additionally, the analysis of body weight changes following challenge highlighted the advantageous effects of probiotic bacteria as vaccine adjuvants.

Intravenous vaccine administration has been investigated since the 1970s. Compared with other immunization approaches, intravenous immunization in experimental mice is associated with (Kemble and Greenberg, 2003) augmented humoral and cellular immune responses (Sun et al., 2013), increased survival rates following challenge, and (Ghafoori et al., 2023) reduced tissue lesions. Our findings demonstrated that at 5 days post-infection (dpi), the counts of B cells, CD4^+^ T cells, and CD8^+^ T cells were significantly higher in mice intravenously administered with inactivated *E. faecium* combined with inactivated H1N1 virus compared to those intramuscularly administered with the same formulation, indicating that intravenous administration enhances adaptive immunity. The analysis of HI assays revealed that intravenous administration of H1N1 was associated with significantly higher viral HI antibody titers compared to intramuscular injection, suggesting that intravenous immunization enhances antibody production. In line with this observation, a study demonstrated that mice inoculated intravenously with BCG cell walls emulsified in Drakeol 6-VR mineral oil exhibited greater resistance to airborne infections caused by the *M. tuberculosis* strain H37Rv than those treated via other routes. Another investigation reported that among seven monkeys vaccinated intravenously with 1 mg of live BCG and subsequently challenged with 40 units of the *M. tuberculosis* strain H37Rv, four showed no gross evidence of disease at autopsy 13 weeks post-infection, while the remaining three animals displayed minimal disease. Compared to intradermal or aerosol immunization, intravenous immunization in rhesus monkeys elicited stronger antigen-specific CD4^+^ and CD8^+^ T cell responses in blood, spleen, lung lymph nodes, and bronchoalveolar lavage. Furthermore, intravenous administration increased the number of antigen-responsive T cells within the lung parenchyma (Anacker et al., 1969; Barclay et al., 1970; Darrah et al., 2020). In humans, the intravenous administration of paraformaldehyde-fixed autologous cells infected *in vitro* with V25 yielded the most effective results for the production of neutralizing antibodies (Zagury et al., 1988). Our previous studies demonstrated that the intravenous delivery of inactivated *E. faecium* and inactivated pseudorabies virus was associated with reduced mortality, enhanced production of viral antibodies, Th cells, Tc cells, as well as increased cytokine expression compared to intramuscular immunization in mice (Cui et al., 2023).

In this study, we observed that HI antibody titers were significantly higher in the intravenous group compared to the intramuscular group (Figure 3). Furthermore, intravenous administration of inactivated *E. faecium* + inactivated H1N1 virus was associated with a greater number of B cells, CD4^+^ T cells, and CD8^+^ T cells at 5 days post-infection (dpi) compared to intramuscular injection (Figure 5), suggesting enhanced adaptive immunity. Body weight measurements and histopathological analyses revealed that intravenous administration of inactivated *E. faecium* + inactivated H1N1 virus was correlated with improved body weight recovery and a more robust immune response (Figures 1, 2), highlighting the benefits of intravenous immunization.

Probiotic bacteria play a crucial role in maintaining immune homeostasis. These microorganisms can translocate from the intestinal mucosa into the bloodstream, thereby exerting systemic effects on various organs, including the lungs (Baud et al., 2020). Probiotics have demonstrated potential for both the prevention and treatment of influenza virus (IV) infections. For example, the oral administration of *Bifidobacterium longum* MM-2 over a period of 17 consecutive days significantly alleviated clinical symptoms, mitigated lung inflammation, reduced mortality rates, decreased viral antibody titers, minimized cell death, and downregulated the expression of pro-inflammatory cytokines in bronchoalveolar lavage fluid. Furthermore, this probiotic strain activated natural killer (NK) cells by upregulating the expression of interferon-gamma (IFN-γ), interleukin-2 (IL-2), interleukin-12 (IL-12), and interleukin-18 (IL-18) in the lungs of mice intranasally inoculated with the H1N1 (PR8) virus. Similarly, the oral or intranasal administration of various Lactobacillus strains has been shown to reduce IV load, enhance salivary and serum IgA levels, increase the number of helper T cells in the lung parenchyma, and upregulate the expression of IFN-γ and tumor necrosis factor-alpha (TNF-α) (Kawahara et al., 2015; Zolnikova et al., 2018). The oral administration of *Lactobacillus delbrueckii* ssp. *bulgaricus* OLL1073R-1 was associated with prolonged survival, attenuated weight loss, reduced viral load, and enhanced production of antiviral antibodies in mice (Nagai et al., 2011; Takahashi et al., 2019). Additionally, the oral administration of a water-soluble fraction derived from heat-treated *E. faecalis* FK-23 resulted in improved survival rates and upregulated expression of the anti-inflammatory cytokine IL-10 in the lungs of mice intranasally challenged with H1N1 influenza virus (Kondoh et al., 2012).

Probiotic bacteria are frequently utilized as vaccine adjuvants to enhance the immunogenicity and efficacy of live vaccines. For example, oral immunization with *E. faecium* L3 has been shown to protect mice against influenza virus (IV), improve survival rates, and mitigate weight loss (Mezhenskaya et al., 2021). In this study, we employed inactivated *E. faecium* as a vaccine adjuvant in combination with IV and evaluated the protective effects of two distinct immunization strategies. The results demonstrated that weight recovery was slower in mice intravenously challenged with live H1N1 virus compared to those challenged with a combination of inactivated *E. faecium* and inactivated H1N1 virus, thereby confirming the probiotic properties of *E. faecium*, which aligns with our previous findings. At 5 days post-infection (dpi), significant differences were observed in the numbers of B cells, CD4^+^ T cells, and CD8^+^ T cells in the spleens of mice intravenously challenged with either live H1N1 virus or a combination of inactivated *E. faecium* and inactivated H1N1 virus (Figure 5). These observations indicate that inactivated *E. faecium* alone does not provide effective protection against IV, corroborating our earlier results (Cui et al., 2023).

Other ongoing studies are evaluating the immunomodulatory effects of two vaccines (inactivated *E. faecium* in Freund’s incomplete adjuvant, and a combination vaccine consisting of inactivated *E. faecium*, porcine reproductive and respiratory syndrome virus, and African swine fever virus), as well as the cross-protection potential of inactivated *E. faecium* and inactivated H1N1 against coronavirus infections and tuberculosis. These findings are expected to enhance the clinical utility of these vaccines.

## Data Availability

The original contributions presented in the study are included in the article/supplementary material, further inquiries can be directed to the corresponding authors.
